# Evaluation of Multiple Choice and Short Essay Question items in Basic Medical Sciences 

**DOI:** 10.12669/pjms.301.4458

**Published:** 2014

**Authors:** Mukhtiar Baig, Syeda Kauser Ali, Sobia Ali, Nighat Huda

**Affiliations:** 1 Dr. Mukhtiar Baig, PhD, MHPE, Professor of Clinical Biochemistry, Head of Assessment Unit, Faculty of Medicine, Rabigh, King Abdulaziz University, Jeddah, Saudi Arabia.; 2 Dr. Syeda Kauser Ali, PhD, Associate Professor, Department of Educational evelopment, Aga Khan University, Karachi, Pakistan.; 3 Dr. Sobia Ali, MHPE, Assistant Professor, Department of Medical Education, Liaquat National Medical College, Karachi, Pakistan.; 4 Ms. Nighat Huda, MS in Ed, Associate Professor, Medical Education Department Bahria University Medical & Dental College, Karachi, Pakistan.

**Keywords:** Assessment, MCQ, SEQ, Item analysis

## Abstract

***Objectives:*** To evaluate Multiple Choice and Short Essay Question items in Basic Medical Sciences by determining item writing flaws (IWFs) of MCQs along with cognitive level of each item in both methods.

***Methods:*** This analytical study evaluated the quality of the assessment tools used for the first batch in a newly established medical college in Karachi, Pakistan. First and sixth module assessment tools in Biochemistry during 2009-2010 were analyzed. Cognitive level of MCQs and SEQs, were noted and MCQ item writing flaws were also evaluated.

***Results:*** A total of 36 SEQs and 150 MCQs of four items were analyzed. The cognitive level of 83.33% of SEQs was at recall level while remaining 16.67% were assessing interpretation of data. Seventy six percent of the MCQs were at recall level while remaining 24% were at the interpretation. Regarding IWFs, 69 IWFs were found in 150 MCQs. The commonest among them were implausible distracters (30.43%), unfocused stem (27.54%) and unnecessary information in the stem (24.64%).

***Conclusion:*** There is a need to review the quality including the content of assessment tools. A structured faculty development program is recommended for developing improved assessment tools that align with learning outcomes and measure competency of medical students.

## INTRODUCTION

Assessment is an essential part of the learning process in education. Students perceive it as a dominant motivator to direct and drive their learning.^1^ The method of assessment determines the approach of students towards learning. Students’ are inclined to espouse a surface approach when assessment emphasis is on recall of factual knowledge and students are more likely to adopt a deep approach^2^ if assessment demands higher levels of cognitive abilities. The approach to learning is a dynamic characteristic and is always modified according to students’ perceptions of the learning environment.^3^ It has been reported that one of the most important factor influencing students’ choice of learning approach is the way how assessment is being conducted.^4^^-^^6^

Multiple methods of assessment namely MCQs, SEQs, OSPE and VIVA are commonly used to assess Basic Science knowledge in undergraduate medical education in Pakistan. Multiple choice questions (MCQs) are the most frequently used type of tests deployed on their own or in combination with other types of test tools for assessment. Moreover, MCQs are appropriate for measuring knowledge, comprehension and could be designed to measure application and analysis.^7^ MCQs are being used increasingly due to their higher reliability, validity, and ease of scoring.^8^^,^^9^ Essay-type assessment is a sensitive test requiring students not only to recall facts but also to use higher-order cognitive skills.^10^ Essay questions though time consuming provides a unique evaluation tool particularly suited for the undergraduate settings.^11^

The use of multiple formats is recommended in assessment of medical students.^12^ However, assessment tools should be valid and reliable and be able to measure the different aspects of professional competencies. The present study was undertaken to evaluate MCQ and SEQ items in Basic Medical Sciences (Biochemistry) by determining item writing flaws (IWFs) of MCQs along with cognitive level of each item in both methods.

## METHODS

This analytical study was carried out in the department of Biochemistry, in a newly established medical college in Karachi, Pakistan. The first batch was admitted in January 2009 while the undergraduate curriculum has been organized in six limited integrated modules. Multiple assessment methods including short essay questions, MCQs, OSPEs, and orals carrying equal weightage were used for module assessment. The college faculty developed all assessment questions.

The cognitive levels of the assessment tools were analyzed using the Buckwalter’s (Buckwalter et al. 1981)^13^ modification of the Bloom’s taxonomy (Bloom 1956).^14^

**Level** **I:** Include questions which attempt to check recall of information.

**Level** **II:** Include questions which attempt to test understanding and interpretation of data.

**Level III** **:** Include questions which attempt to test the application of knowledge for solving a particular problem.

For determining types of item writing flaws (IWFs) standard criteria given by Case and Swanson (2003), Haladyna et al., (2002) & Tarrant et al., (2008) were used and 14 commonly occurring violations of item-writing guidelines were identified.^8^^,^^15^^,^^16^

One subject expert and three medical educationists reviewed each assessment tool. Initially the reviewers individually reviewed the assessment tool for the cognitive level and IWFs according to predefined criteria and reported their results to the principal investigator. A consensus meeting was called to reach on unanimous decision about the debatable questions.

## RESULTS

A total 150 of MCQs that were administered in all six module examinations were reviewed. The cognitive level of 114 MCQs (76%) was at recall level while remaining 36 MCQs (24%) were of interpretation of data and there was no MCQ evaluating problem solving domain of knowledge (Table-I). A total of 36 SEQs were administered in all six module examinations. The cognitive level of 30 SEQs (83.33%) was assessing recall of knowledge while remaining 6 SEQs (16.67%) were assessing interpretation of data and there was no SEQ assessing problem solving domain of knowledge (Table-I). 

A total of 150 MCQs were administered in the all six module examinations. Upon review, 69 IWFs were found and four most common IWFs were implausible distracters (30.43%), unfocused stem (27.54%), unnecessary information in the stem (24.64%), and negative stem (8.7%), respectively (Table-II).

## DISCUSSION

The selection of an appropriate assessment method for measuring students’ performances remains a daunting task for many medical institutions in Pakistan. Attempts to change existing assessment methods have been hindered by financial constraints, lack of expertise in psychometric analysis of the examination and institutional policies. 

The present research found that 76% MCQs were testing the recall of isolated facts while remaining 24% MCQs were testing the skill of interpretation of data and there was not a single MCQ assessing the higher cognitive domains of application and analysis. It may be due to the fact that MCQs at recall level are easier to construct and need less time and knowledge as compare to problem solving MCQs which needs expertise and training.^8^^,^^9^

Khan and Aljarallah (2011)^17^ in their study found that the percentage of MCQs testing the level III (problem solving) cognitive skills of the students was 60%, level II (interpretation of data) was 6% and level I recall of information was 28%. But in that study a total of 50 MCQs representing different disciplines of medicines like gastroenterology, cardiology, neurology, rheumatology, nephrology etc were evaluated. Constructing problem solving MCQs in basic sciences is difficult in comparison to clinical sciences. In present study, multiple factors have contributed to low cognitive level questions such as newly established medical college with inadequate faculty training, diverse background of teachers, non-existence of question bank, first examination of the college etc. Tarrant and Ware (2008) ^16^ found in a nursing examination that over 90% of MCQs were written at low cognitive levels, and that MCQs written at a lower cognitive level were significantly more likely to contain item-writing flaws. Jozefowicz et al. (2002)^18^ evaluated the quality of in-house developed examinations at three US medical schools and found that the overall quality of the questions used was low. Several studies have confirmed that MCQs not only test the knowledge of the examinees but can also be used for measuring higher cognitive skills.^19^^,^^20^

One of the most common problems affecting MCQs quality is the presence of item writing flaws. Item-writing flaws (IWFs) are violations of these accepted item-writing guidelines which can affect student performance on MCQs, making the item either easier or more difficult.^21^ The present study found 69 IWFs (46%) in total 150 MCQs in Biochemistry module exams, and four most common IWF were implausible distracters (30.43%), unfocused stem (27.54%), unnecessary information in the stem (24.64%), and negative stem (8.7%). These results are in agreement with several studies.^16^^,^^20^^,^^22^^,^^23^

Another study documented that 12% of their exam MCQs had item writing flaws.^17^ But in that study all MCQs belonged to clinical sciences subjects, and 60% of the MCQs assessed students’ problem solving skills. In our study problem solving MCQs were zero% and 76% of the MCQs assessed recall of isolated facts. Our higher percentages IWFs can be explained in the view of Tarrant and Ware (2008) study who concluded that MCQs written at lower cognitive levels, are more likely to contain item-writing flaws.^16^ Downing (2005)^21^ assessed the quality of four examinations given to medical students in the United States of America, and found that 46% of MCQs contained IWFs and reported that as a consequence of these IWFs, 10–15% of students who were classified as failures would have been classified as pass if items with IWFs were removed.

**Table-I:** Distribution of cognitive levels of SEQs and MCQs in all six modules. **(**n= Number of SEQs, and MCQs in each module)

* Module*	*Cognitive levels of SEQs* *(n=6)*			*Cognitive levels of MCQs* *(n=25)*		
	C1	C2	C3	C1	C2	C3
M1	6	0	0	24	1	0
M2	4	2	0	18	7	0
M3	6	0	0	20	5	0
M4	5	1	0	20	5	0
M5	5	1	0	16	9	0
M6	5	1	0	16	9	0
Total	31	5	0	114	36	0
Percentage	86.11%	13.89%	0%	76%	24%	0%

C1= Cognitive level 1, C2= Cognitive level 2, C3= Cognitive level 3,

**Table-II:** Frequency of types of IWFs in MCQs in all six modules

*Types of IWFs*	*M1*	*M2*	*M3*	*M4*	*M5*	*M6*	*Total*	*%*
Absolute terms	0	0	0	0	0	0	0	00
Vague terms	0	0	0	0	0	0	0	00
Implausible distractors	11	4	1	1	1	3	21	30.43
Extra details in correct option	1	0	0	0	0	0	1	1.44
Unfocused stems	1	5	5	2	2	4	19	27.54
Grammatical clues	0	0	0	0	0	0	0	00
Logical clues	0	3	0	0	0	0	3	4.35
Word repeats	0	0	1	0	0	0	1	1.44
> 1 correct answer	0	0	0	0	0	0	0	00
Unnecessary information in stem	2	6	3	3	2	1	17	24.64
Lost sequences in data	1	0	0	0	0	0	1	1.44
All of the above	0	0	0	0	0	0	0	00
None of the above	0	0	0	0	0	0	0	00
Negative stem	5	0	0	1	0	0	6	8.7
Total	21	18	10	07	05	8	69	

Results of present study showed presence of 46% of flawed items which could be mostly attributed to insignificant faculty development programs. Flawed items affect difficulty and discrimination index. Low difficulty and poor discrimination in an item favors low achievers while higher difficulty and poor discrimination negatively affected the high scorers and moreover flawed items also fail to assess the course learning objectives.^23^

For reducing IWFs, and improving cognitive levels of the test items, Downing 2006, suggested the use of test blue print.^24^ A blue print is simply a grid or table that maps the course objectives and content to be tested, and is an essential step in generating a valid and reliable test. Test blue print helps in accurately delineating the percentage of test questions to be allocated to the different content areas, and at different cognitive levels.

Faculty should be encouraged, and trained to construct MCQs for higher order cognitive levels. Tarrant et al., (2006)^25^ pointed out that by removing IWFs from MCQs does not necessarily change the cognitive domain of a question, but writing questions at higher cognitive levels inherently remove numerous IWFs.

The present study suggest that there is need to improve the quality of our assessment tools because if the assessment tools measur low cognitive level, it will not only decrease the validity of the exam but also compel the students to adopt surface learning approaches which is not suitable for lifelong learning.

***Limitations of the study: ***The study analyzed results of only two modules, and students’ scores in only one subject. Moreover, difficulty and discrimination indices were not available.

## CONCLUSION

The medical college should evolve policy guidelines on preparing questions of higher cognitive level for all departments and student assessment should align with the learning outcomes. There is need to review the quality of item including the content of assessment tools.

## Authors Contribution:

**MB: **Designed the study, did all analysis and prepared the manuscript.

**KA, SA, NH: **Helped in evaluating the quality of the assessment tools, drafting and revising the manuscript.

## Notes

***Recommendations: ***Psychometric analysis should be done for all types of assessment while test blue print should be developed to ensure validity. A structured faculty development program is recommended for developing improved assessment tools. Similar studies are recommended in other basic science subjects.

## References

[1] Drew S (2001). Student perceptions of what helps them learn and develop in higher education. Teaching Higher Educ.

[2] Scouller K (1998). The influence of assessment method on students’ learning approaches: multiple-choice question examination versus assignment essay. Higher Educ.

[3] Struyven K, Dochy F, Janssens S (2005). Students’ perceptions about evaluation and assessment in higher education: a review. Assess Evaluation Higher Educ.

[4] Trigwell K, Prosser M (1991). Improving the quality of student learning: the influence of learning context and student approaches to learning on learning outcomes. Higher Educ.

[5] Biggs J (2003). Teaching for Quality Learning at University.

[6] Reid WA, Duvall E, Evans P (2007). Relationship between assessment results and approaches to learning and studying in Year Two medical students. Med Educ.

[7] Abdel-Hameed AA, Al-Faris EA, Alorainy IA (2005). The criteria and analysis of good multiple choice questions in a health professional setting. Saudi Med J.

[8] Case S, Swanson D (2003). Constructing written test questions for the basic and clinical sciences.

[9] Tarrant M, Ware JA (2012). Framework for improving the quality of multiple-choice Assessments. Nurse Educator.

[10] Pepple DJ, Laurine YE, Carroll RG (2010). A comparison of student performance in multiple-choice and long essay questions in the MBBS stage I physiology examination at the University of the West Indies (Mona Campus). Adv Physiol Educ.

[11] Oyebola DO, Adewoye OE, Iyaniwura J O, Alada RA, Fasanmade AA, Raji YA (2000). Comparative study of students’ performance in preclinical physiology assessed by multiple choice and short essay questions. Afr J Med Sci.

[12] Epstein MR (2007). Assessment in Medical Education. N Engl J Med.

[13] Buckwalter JA, Schumacher R, Albright JP (1981). Use of an educational taxonomy for evaluation of cognitive performance. J Med Educ.

[14] Bloom B, Englehart M, Furst E (1956). Taxonomy of educational objectives: The classification of educational goals. Handbook I: Cognitive domain.

[15] Haladyna TM, Downing SM, Rodriguez MC (2002). A review of multiple-choice item writing guidelines. Applied Meas Educ.

[16] Tarrant M, Ware J (2008). Impact of item-writing flaws in multiple choice questions on student achievement in high-stakes nursing assessments. Med Educ.

[17] Khan MZ, Aljarallah BM (2011). MEQs and MCQ as a tool for assessing the cognitive skills of undergraduate medical students. Int J Heal Sci.

[18] Jozefowicz RF, Koeppen BM, Case S (2002). The quality of in-house medical school examinations. Acad Med.

[19] Mukhopadhyay M, Bhowmick M, Chakraborty S (2010). Evaluation of MCQs for judgment of higher levels of cognitive learning. Gomal J Med Sci.

[20] Palmer EJ, Devitt PG ( 2007). Assessment of higher order cognitive skills in undergraduate education: modified essay or multiple choice questions?. BMC Med Educ.

[21] Downing SM (2005). The effects of violating standard item writing principles on tests and students: the consequences of using flawed test items on achievement examinations in medical education. Adv Health Sci Educ.

[22] Downing SM ( 2002). Construct-irrelevant variance and flawed test questions: do multiple-choice item-writing principles make any difference?. Acad Med.

[23] Al Muhaidib NS (2010). Types of Item-Writing Flaws in Multiple Choice Question Pattern- A comparative study. J Educ Psychol Sci.

[24] Downing SM, Downing SM, Haladyna TM (2006). Twelve steps for effective test development. Handbook of test development.

[25] Tarrant M, Knierim A, Hayes SK (2006). The frequency of item writing flaws in multiple-choice questions used in high stakes nursing assessments. Nurse Educ Pract.

